# Immunoenhancement Function of the Novel Hexapeptide (LVVLGH) from Thick-Shelled Mussel (*Mytilus coruscus*) on Immunodeficient Mice by Activating the NF-κB/MAPK Pathway

**DOI:** 10.3390/foods14111865

**Published:** 2025-05-24

**Authors:** Xu Yang, Yu Zeng, Fandi Que, Shiqing Fu, Li Xu, Fangmiao Yu, Bin Wang

**Affiliations:** Zhejiang Provincial Engineering Technology Research Center of Marine Biomedical Products, School of Food and Pharmacy, Zhejiang Ocean University, Zhoushan 316022, China; yangxu7150202505@163.com (X.Y.); 18358590405@163.com (S.F.); wangbin@zjou.edu.cn (B.W.)

**Keywords:** thick-shelled mussel (*Mytilus coruscus*), peptides, Cyclophosphamide, immunomodulatory effect, LVVLGH (LH-6), NF-κB/MAPK pathway

## Abstract

A novel hexapeptide LVVLGH (LH-6) from the thick-shelled mussel (*Mytilus coruscus*) demonstrated potent immune-enhancing effects in RAW264.7 cells in vitro, but its immunological activity in vivo is unclear. As a result, the present study was designed to investigate the in vivo effects of LH-6 on cyclophosphamide-induced immunodeficient mice. The results demonstrate that LH-6 promoted the growth and development of immunodeficient mice in a concentration-dependent manner, remarkably elevated the immune organ index, and relieved the pathological characteristics of the spleen and thymus. Additional experiments also revealed that LH-6 effectively promoted the multiplication of splenic lymphocytes and natural killer activity, enhanced the function of abdominal macrophages, and apparently recovered delayed-type hypersensitivity in immunodeficient mice. The secretion of IgA, IgG, IgM, TNF-α, IL-1β, IL-6, and serum hemolysin were remarkably improved by LH-6, suggesting that LH-6 can synergistically strengthen cellular and humoral immunity. In addition, LH-6 promoted the phosphorylation of IκBα and nuclear translocation of p65, which correspondingly increased the phosphorylation levels of p38, JNK, and ERK; activated the NF-κB and MAPK pathways; and exerted in vivo immunomodulatory activities. Docking results show that LH-6 has favorable binding energies to candidate proteins in the NF-κB and MAPK pathways. To summarize, this research further demonstrated that LH-6 possesses in vivo immunomodulatory activity, which provides a possibility for the subsequent development of immune-enhancing functional foods.

## 1. Introduction

The immune system is a complex functional system of various organs, tissues, cells, and cytokines to defend the host from external pathogen infections and tumors [1,2]. Immune function is susceptible to a variety of adverse factors, including environmental pollution, dietary habits, and side effects of medications, and a dysfunctional immune system increases the risk of infection and cancer. Therefore, boosting immunity to enhance immune function is essential for the prevention of disease and the recovery of health. Cyclophosphamide (CTX) is frequently utilized in cancer therapy as a DNA cross-linking agent that alkylates DNA, which inhibits the growth and reproduction of tumor cells [3]. Meanwhile, CTX has a significant inhibitory effect on lymphocytes, which can lead to immunodeficiency with long-term use [4].Currently, chemically synthesized drugs such as levamisole [5], interferon [6], interleukin (IL)-2 [7], and Bacille Calmette–Guérin (BCG) vaccine [8] are extensively used as immunomodulators in clinical therapy, but side effects arising from their usage, including nausea, vomiting, and headache, are still unavoidable. To address this issue, it is significant for researchers to seek novel low-toxicity and effective immunomodulators that can regulate the immune function.

In recent years, the utilization of naturally derived bioactive peptides as functional foods or immunomodulators to replace chemically synthesized drugs has become a focus of researchers in an attempt to reduce the toxic adverse effects of regular treatments [9]. As marine organisms are exposed to extremely harsh living environments, they produce marine peptides containing special structures that have been demonstrated to have a wealth of biological activities [10], including antimicrobial [11], anti-fatigue [12], antithrombotic [13], anti-osteoporosis [14], antihypertensive [15,16], antitumor [17], antioxidant [18], and immunomodulatory activities [19]. Among them, a multitude of successful isolations of immunomodulatory peptides from marine organisms has been reported. For example, low-molecular-weight oyster peptides can reduce spleen damage, increase the number of splenic T-lymphocytes and immunoglobulin secretion, and ameliorate the composition of intestinal flora in CTX-induced immunodeficient mice, thereby enhancing overall immunity [20]. Ethanol-soluble oligopeptides (CP-ES) from *Gadus morhua* not only promoted the proliferation of mice splenic lymphocytes and T-lymphocytes and enhanced cellular immunity, but also interacted with Toll-like receptor 2 (TLR2) to facilitate the gene expression of RAW264.7 cell-associated cytokines to play the role of immunomodulation [21]. Correspondingly, a pentadecapeptide (RVAPEEHPVEGRYLV) isolated from *Cyclina sinensis* strengthened the immunity of immunodeficient mice by upregulating the secretion of pro-inflammatory factors through the MAPK/NF-κB and PI3K/Akt pathways [22]. The safety, bioavailability, and various pharmacological activities of these peptides offer substantial potential for functional food discovery and applications in other fields.

Mussels are common mollusks in the ocean. Among them, thick-shelled mussel (*Mytilus coruscus*) is a bivalve species, which is extensively distributed along the eastern coast of China and favored by people because of its tasty and nutritious meat [23]. To date, thick-shelled mussel peptides have attracted increasing focus due to their various biofunctions, such as antioxidant activity [24], antimicrobial activity [25], and hepatoprotective effect [26]. According to our previous study, the low-molecular-weight *M. coruscus* protein hydrolysate (MCP) was found to have significant proliferation and phagocytosis effects on macrophages [27], and confirmed that the key active peptide in MCP, LH-6, had a marked immunomodulatory effect in vitro [28]. Nonetheless, studies on the impact of thick-shelled mussel peptides on immunomodulatory activity in vivo are still lacking. Therefore, the present study systematically investigated the in vivo immunomodulatory activity of LH-6 in CTX-induced immunosuppressed mice from the perspective of cellular and humoral immunity.

## 2. Materials and Methods

### 2.1. Materials, Reagents, and Instruments

LVVLGH (LH-6) with a purity of 98.67% was synthesized by Shanghai Apeptide Co., Ltd. (Shanghai, China). ConA and CTX were purchased from Shanghai Yuanye Biotechnology Co., Ltd. (Shanghai, China). Roswell Park Memorial Institute 1640 (RPMI 1640) was obtained from Gibco (Shanghai, China). YAC-1 and guinea pig serum were provided by SenBeiJia Biological Technology Co., Ltd. (Nanjing, China). Sulfosalicylic acid (SA) buffer was purchased from Beijing Baiaolaibo Technology Co., Ltd. (Beijing, China). BCA, nitric oxide (NO), LDH, CCK-8, neutral red, assay kits, and all the primary antibodies against were all supplied by Beyotime Biotechnology (Shanghai, China).

### 2.2. Animals and Treatments

**Figure 1:**
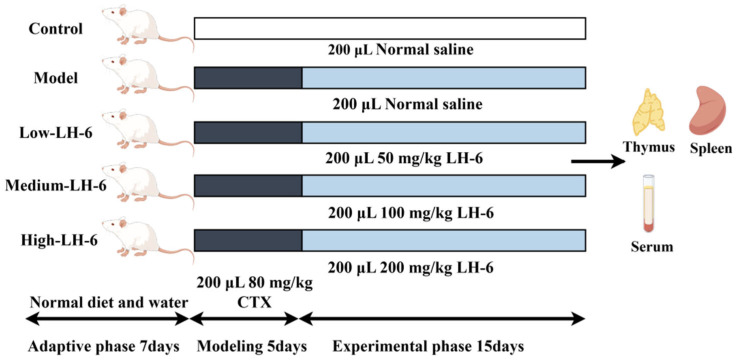
Experimental arrangement of mice in this study (*n* = 10).

Specific-pathogen free ICR mice were purchased from Zhejiang Experimental Animal Center (Hangzhou, China), and the animal experiments were authorized by the Zhejiang Ocean University Ethics Committee. Immunosuppressed mice were established based on the method of Yang et al. [29]. The experimental protocol shown in Figure 1 was drawn by Figdraw. After 7 days of acclimatization feeding, 50 male ICR mice (6–7 weeks old, 22–24 g) were randomly divided into 5 groups (*n* = 10 per group). The control group was intraperitoneally injected with 0.9% saline for 5 days, and the model and experimental groups were intraperitoneally injected with CTX 80 mg/kg for 5 days, once a day. Then, the experimental groups were gavaged with different concentrations of LH-6 (50, 100, 200 mg/kg/d), and the model group and normal group were gavaged with 0.9% saline, all for 15 days.

### 2.3. Measurement of Body Weight and Thymus and Spleen Index

After acclimatization feeding was completed, the mice were weighed every two days. After the mice were executed using the cervical dislocation method, the thymus and spleen were collected and weighed [30]. The thymus and spleen indexes were obtained as follows:

Thymus or spleen index (mg/g) = thymus or spleen weight (mg)/body weight (g) × 100%

### 2.4. H&E Staining of Thymus and Spleen

The removed thymus and spleen tissue were fixed with 4% paraformaldehyde for 24 h, washed with running water overnight, and paraffin sections were prepared and stained [31]. After staining, pictures were taken under an optical microscope (biological microscope CX31, Olympus, Tokyo, Japan).

### 2.5. Assay of Splenic Lymphocyte Proliferation

Spleens were dissected aseptically, and splenic tissue was sheared and placed in a 200-mesh cell strainer containing 5 mL PBS. The single-cell suspension was milled and filtered with a syringe stopper and centrifuged at 100× *g* for 5 min. An aliquot of 2 mL 1× red cell lysate was added, resuspended, and allowed to stand for 2 min; terminated with 10 mL PBS; and centrifuged again. An aliquot of 2 mL RPMI-1640 medium was added, resuspended, and counted, and the cell number was adjusted to 3 × 10^6^ cells/mL. For each group of splenic lymphocytes, a control group (100 μL cell suspension + 100 μL nutrient solution) and a ConA group (100 μL cell suspension + 100 μL 5 μg/mL ConA) were set up, and incubation was continued for 8 h. The proliferation rate of the cells in each group was detected using the CCK-8 kit. The proliferative capacity of the splenocytes was calculated as follows (1):

Splenocyte proliferation capacity = OD_ConA_/OD_control_ × 100% (1)

### 2.6. Determination of Phagocytosis and NO Synthesis in Macrophages

Mouse macrophages were extracted by a method slightly different from that of Zhang et al. [32]. After the mice were executed by cervical dislocation method, they were immersed in 75% ethanol for 5 min. In a sterile environment, the abdominal skin was peeled off, 2 mL pre-cooled PBS was injected into the abdominal cavity, the position of the syringe was fixed, and the liquid in the abdominal cavity was sucked out with the original syringe by rubbing the syringe with the fingertips for 1–2 min. The aspirated liquid was centrifuged at 100× *g* for 5 min, resuspended with 2 mL PBS, centrifuged again, and repeated twice before 2 mL RPMI-1640 medium was added and resuspended for counting. Macrophages were inoculated into 96-well plates and incubated for 4 h. At the end of the incubation, neutral red phagocytosis and NO synthesis were measured in mouse peritoneal macrophages.

### 2.7. Assay of DTH

Measurement of delayed-type hypersensitivity (DTH) was performed using the metatarsal thickening method according to Pasala et al. [33]. A total of 200 μL of 2% (*v*/*v*) sheep red blood cell (SRBC) was injected intraperitoneally, and the thickness of the left hindfoot was measured 4 days later and re-injected at the same site. The thickness of the site was measured three times after 24 h, and the value of the difference between the pre- and post-measurements was calculated.

### 2.8. Measurement of Mouse Serum Hemolysin Half Hemolysin Value (HC_50_)

HC_50_ was determined by referring to and appropriately modifying the method of Gao et al. [34]. Six mice from each group were taken and injected intraperitoneally with 200 μL of 2% (*v*/*v*) SRBC in the morning. Four days later, blood was taken from the orbits and centrifuged at a low temperature to collect serum. The serum was diluted with SA buffer at 100-fold and guinea pig serum was diluted with SA at 1:8 to obtain complement. The blank group (100 μL SA + 50 μL 10% (*v*/*v*) SRBC + 100 μL complement), the experimental group (100 μL diluted serum + 50 μL 10% (*v*/*v*) SRBC + 100 μL complement), and the SRBC group (100 μL 10% (*v*/*v*) SRBC + 900 μL ultrapure water) were set up for a 37 °C water bath. The OD value of the supernatant was measured at 540 nm and the HC_50_ was calculated according to the following Formula (2):

HC_50_ = (OD_experimental group_ − OD _blank group_)/(OD_SRBC_ − OD _blank group_) × 100% (2)

### 2.9. Assay of Natural Killer (NK) Cells

The assay of mouse NK cell activity was slightly modified from the procedure of Jang et al. [35]. The target cells, mouse lymphoma cells (YAC-1), were cultured for 24 h, and mouse splenic lymphocytes were used as effector cells. The 96-well plate was set up with a natural release group (100 μL YAC-1 + 100 μL nutrient solution), a maximal release group (100 μL YAC-1 + 100 μL LDH-releasing reagent), and experimental groups (100 μL splenocytes + 100 μL YAC-1, with an efficacy-to-target ratio of 50:1) three times in parallel. The groups were incubated for 1 h, with the OD values measured at 490 nm. NK cell activity was calculated as follows:

NK cell activity = (OD_experimental group_ − OD_natural release_)/(OD_Maximum release_ − OD_natural release_) × 100% (3)

### 2.10. Determination of Cytokine and Immunoglobulins in Serum

Serum concentrations TNF-α, IL-6, IL-1β, IgA, IgM, and IgG were analyzed using an Elisa kit according to the manufacturer’s instructions.

### 2.11. Western Blot Analysis

Referring to the method of Huang et al. [36] with slight modification, spleens from different groups of mice were collected and treated with 1 mL of cell protein lysate for 30 min and centrifuged, and the protein concentration in the supernatant was determined. Then, separating and concentrating gels were prepared. When the electrophoresis was finished, the membrane was turned over to the closed state, and the primary antibody was added and incubated at 4 °C overnight. The membranes were washed three times with Tris Buffered Saline with Tween-20 (TBST), incubated with the corresponding secondary antibodies for 2 h at room temperature, washed three times with TBST, and then developed and analyzed by Alpha view SA software (version 3.4.0) (Fluor Chem FC3, Fluor Chem, Irving, TX, USA).

### 2.12. Molecular Docking Analysis

Molecular docking was utilized to assess the affinity of LH-6 for specific proteins of NF-κB and MAPK. The receptor proteins MyD88 (PDB ID: 7BEQ), ERK1 (PDB ID: 2ZOQ), and JNK1 (PDB ID: 4L7F) were downloaded from the RCSB protein database (https://www.rcsb.org/) (11 December, 2024). After removal of water molecules, molecular docking of LH-6 and the corresponding target proteins was performed using HPEPDOCK. Ligand–receptor complexes were visualized using Maestro 13.9 academic software.

### 2.13. Statistical Analysis

The experimental data were expressed as mean ± standard deviation (X ± SD), and one-way ANOVA was performed with SPSS 26.0, the Duncan test was completed for significant differences, and *p* < 0.05 was considered statistically significant.

## 3. Results

### 3.1. Effect of LH-6 on Body Weight and Thymus and Spleen Index

**Figure 2:**
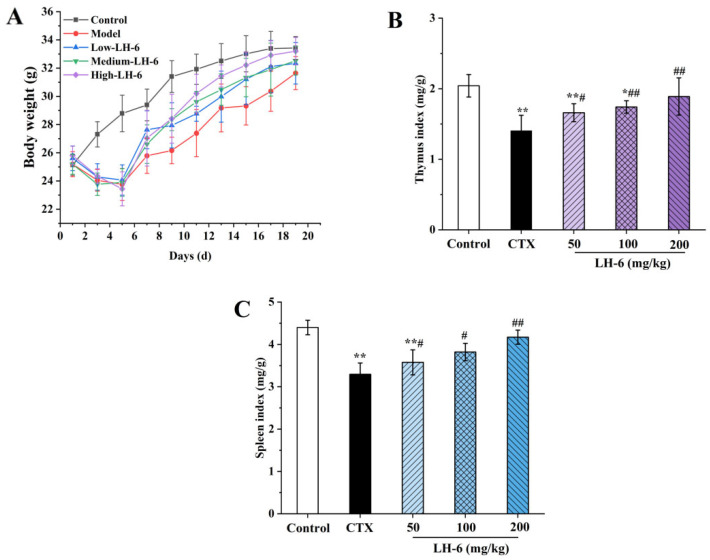
Effect of LH-6 on body weight variation and immune organ indices in CTX-induced mice. (**A**) Body weight; (**B**) thymus index; (**C**) spleen index. 5 groups, *n* = 10. * *p* < 0.05 and ** *p* < 0.01 vs. control group; # *p* < 0.05 and ## *p* < 0.01 vs. model group.

As shown in Figure 2A, during the modeling period, mice injected intraperitoneally with CTX lost a significant amount of body weight in the first five days, whereas the control group remained on an upward trend, indicating the successful construction of immunosuppressed mice. After the administration of different doses of LH-6, the weight of the LH-6 group was elevated in a time-dependent manner, and the growth in body weight was increased more than in the model group, in which the body weights of mice in the 200 mg/kg LH-6 group gradually approached those of the control group, which indicated that LH-6 could ameliorate CTX-induced developmental disorders in mice.

The thymus and spleen are essential constituents of the immune system, with their status being a good indicator of resistance to exogenous pathogens [37]. Figure 2B,C shows the effects of LH-6 on the indices of immune organs in mice. The indices of the thymus (Figure 2B) and spleen (Figure 2C) in the model group were 1.40 ± 0.22 and 3.29 ± 0.27, which were noticeably lower than those of normal mice (2.04 ± 0.16 and 4.40 ± 0.17) (*p* < 0.01), suggesting that CTX induced atrophy of immune organs. In comparison with the model group, a concentration-dependent increase in thymic and splenic indices was observed in LH-6-treated immunosuppressed mice, and the thymus and spleen indices of the 200 mg/kg LH-6 group were extremely significant (*p* < 0.01), demonstrating that LH-6 has a certain repairing effect on CTX-induced damage to immune organs.

### 3.2. Histopathologic Analysis of Thymus and Spleen

**Figure 3:**
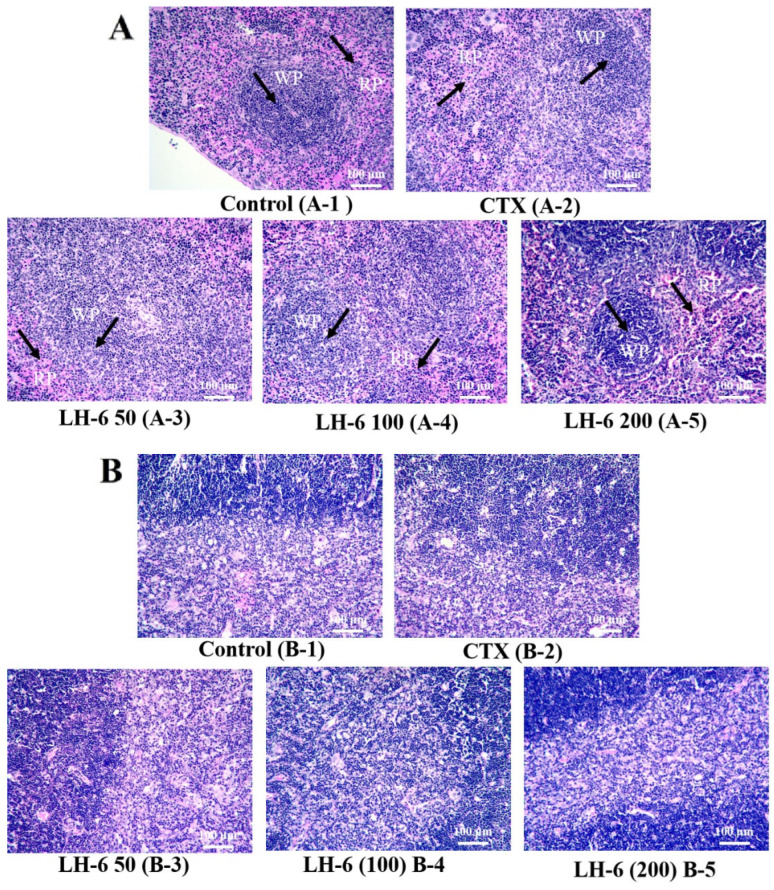
H&E staining results of mouse spleen and thymus. (**A**) HE-stained spleen tissues (200×), WP: white pulp, RP: red pulp; (**B**) HE-stained thymus tissues (200×).

Figure 3 depicted the morphologic observation of LH-6 recovery from spleen and thymus injury (File S1). The spleen is mainly composed of red pulp, white pulp, and a marginal zone [38]. Figure 3A shows that the spleen in the model group was structurally disorganized, with blurred boundaries between red and white pulp and dispersed splenocytes, as compared to the control group [9]. The administration of LH-6 alleviated these conditions, particularly in the 200 mg/kg LH-6 group, with clearer boundaries between red and white pulp. In addition, as shown in Figure 3B, it was observed that the borders of the thymic cortex and medulla were not sharply defined, the number of thymocytes was reduced, and the lymphocytes were loosely arranged in the model group [39]. After the administration of LH-6, the boundary between the thymic cortex and medulla became distinct and the cortex thickened. Histopathologic observations revealed that LH-6 restored CTX-induced splenic and thymic injuries in a dose-dependent manner.

### 3.3. Effect of LH-6 on Spleen Lymphocytes

**Figure 4:**
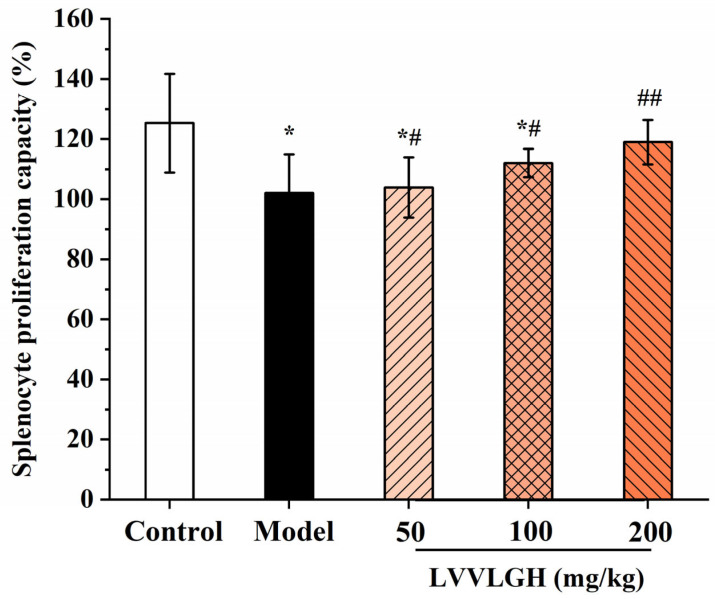
In vitro proliferation ability of mice spleen lymphocytes. 5 groups, *n* = 10. * *p* < 0.05 vs. control group; # *p* < 0.05 and ## *p* < 0.01 vs. model group.

The proliferative activity of lymphocytes was determined to assess the effect of LH-6 on cellular immune response. Figure 4 shows that the proliferative activity of splenic lymphocytes was significantly lower in the model group (102.07 ± 12.93%) compared to the control group (125.34 ± 16.44%) (*p* < 0.05). However, splenic lymphocyte activity in the peptide group was positively correlated with the concentration of LH-6, especially in the 200 mg/kg LH-6 group, where the proliferative activity reached 118.99 ± 7.39% (*p* < 0.01).

### 3.4. Effect of LH-6 on Phagocytic Activity and NO Secretion in Peritoneal Macrophages

**Figure 5:**
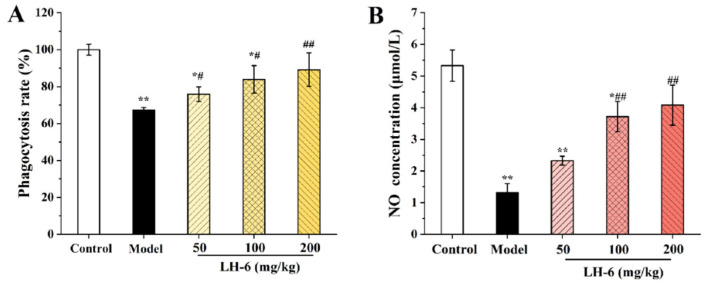
The phagocytosis (**A**) and NO secretion (**B**) of mice peritoneal macrophages 5 groups, *n* = 10. * *p* < 0.05 and ** *p* < 0.01 vs. control group; # *p* < 0.05 and ## *p* < 0.01 vs. model group.

Macrophages, a key member of innate immunity, phagocytose damaged cells and pathogens and play a crucial role in maintaining immune homeostasis [40]. The effect of LH-6 on the phagocytic activity of peritoneal macrophages in immunosuppressed mice is determined by neutral erythrocyte quantification. As shown in Figure 5A, compared to the control group, the phagocytic rate in the model group (67.38 ± 1.27%) was remarkably lower. It is worth noting that peritoneal macrophage phagocytosis rates were 75.91 ± 3.93%, 83.90 ± 7.50%, and 89.16 ± 9.06% in the low-, medium-, and high-dose LH-6 groups, respectively, which were higher than those in the model group but still lower than those in the control group (*p* < 0.05). These results exhibit that LH-6 significantly enhanced the immunological activity of mouse peritoneal macrophages.

NO is an important biomessenger and effector molecule produced by immune cells to exert various physiological events, including killing microorganisms and tumor cells; modulating the secretion of cytokines, chemokines, and growth factors; and messaging [41,42]. As illustrated in Figure 5B, a noticeable decrease (*p* < 0.01) in NO secretion was observed in the model group, which was only 1.32 ± 0.28 μmol/L compared with the control group. In contrast to the model group, LH-6 administration promoted macrophage NO secretion, and the NO content was 3.72 ± 0.48 and 4.08 ± 0.63 μmol/L in the medium- and high-concentration LH-6 groups, respectively, with a remarkable increase in NO secretion (*p* < 0.01).

### 3.5. Effect of LH-6 on DTH in Mice

Cellular immune function could be assessed by measuring toe thickening, and the results are shown in Table 1. The toe swelling was obviously decreased in the model group (0.26 ± 0.03 mm) in comparison to the control group (0.51 ± 0.07 mm) (*p* < 0.01). It is notable that though there was no distinct difference among the 50 mg/kg LH-6 group and the model group, the difference was noticeable with respect to the 200 mg/kg LH-6 group (0.43 ± 0.11 mm) (*p* < 0.01), which indicated that the toe thickening of the mice in the LH-6 group was positively proportional to the dose, restored the delayed hypersensitivity, and improved the cellular immune function of the mice.

### 3.6. Effect of LH-6 on the Humoral Immune Response and NK Cell Killing Activity

**Figure 6:**
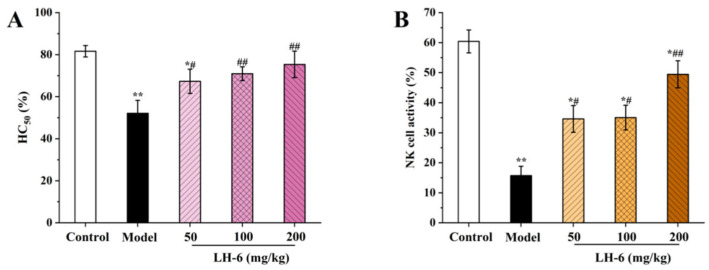
The effect of LH-6 on HC_50_ (**A**) and NK cell activity (**B**) in mice. 5 groups, *n* = 10. * *p* < 0.05 and ** *p* < 0.01 vs. control group; # *p* < 0.05 and ## *p* < 0.01 vs. model group.

Figure 6A shows the changes in serum hemolysin in mice. As compared to the control group (81.63 ± 2.72%), the HC_50_ value of the model group (52.11 ± 6.14%) was significantly decreased (*p* < 0.01), which indicates that CTX reduced the complement content in the serum of mice. In comparison with the model group, the HC_50_ values were all apparently increased to 67.30 ± 5.77%, 70.95 ± 3.28%, and 75.38 ± 6.31% in the low-, medium-, and high-dose groups of LH-6, which were positively correlated with the concentration. This suggests that LH-6 can improve the low level of serum hemolysin induced by CTX in mice, enhance the immunomodulatory function, and increase the serum antibody to SRBC protein.

Figure 6B shows the changes in NK cell activity under the effect of LH-6. Compared with the control group, the NK cell activity in the model group was only 15.73 ± 3.10%, and the cell activity was significantly reduced (*p* < 0.01). After treatment with LH-6, the NK cell activities were 34.59 ± 4.44%, 35.05 ± 4.13%, and 49.46 ± 4.52% in the low-, medium-, and high-dose groups of LH-6, respectively, in which the activity of the 200 mg/kg LH-6 group was distinctly enhanced as compared with the model group (*p* < 0.01). The results demonstrate that LH-6 enhanced the activity of NK cells in immunocompromised mice and improved the immunity of the organism.

### 3.7. Effect of LH-6 on Serum Cytokines

**Figure 7:**
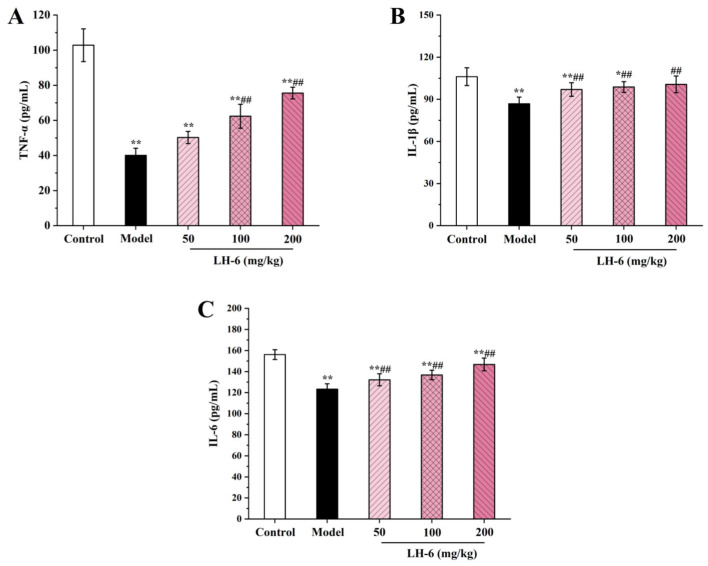
The effect of LH-6 on serum TNF-α (**A**), IL-1β (**B**), and IL-6 (**C**) levels in mice. 5 groups, *n* = 10. * *p* < 0.05 and ** *p* < 0.01 vs. control group; ## *p* < 0.01 vs. model group.

Cytokines including TNF-α, IL-1β, and IL-6 are secreted mainly by macrophages and lymphocytes, linking innate and acquired immunity for mediating the release of immune responses [43]. In Figure 7A, TNF-α was dose-dependently elevated in the serum of mice after treatment with LH-6. In particular, the TNF-α levels in the 100 and 200 mg/kg LH-6 groups were 62.38 ± 6.83 and 75.56 ± 3.31 pg/mL, and were substantially above those of the model group (40.12 ± 3.99 pg/mL). In Figure 7B,C, the IL-1β levels in the 100 and 200 mg/kg LH-6 groups were 98.80 ± 3.82 and 100.63 ± 5.94 pg/mL, and were notably more favorable than those in the model group (86.83 ± 4.77 pg/mL) (*p* < 0.01). Correspondingly, the IL-6 levels in the 100 and 200 mg/kg LH-6 groups were 136.81 ± 4.50 and 146.79 ± 6.06 pg/mL, and were also considerably above those of the model group (123.33 ± 5.12 pg/mL) (*p* < 0.01). However, serum levels of TNF-α, IL-1β, and IL-6 were still lower in the LH-6 group than in the control group. These results suggest that LH-6 can counteract a partial reduction in serum cytokines in mice caused by CTX.

### 3.8. Effects of LH-6 on Serum Immunoglobulins Levels

**Figure 8:**
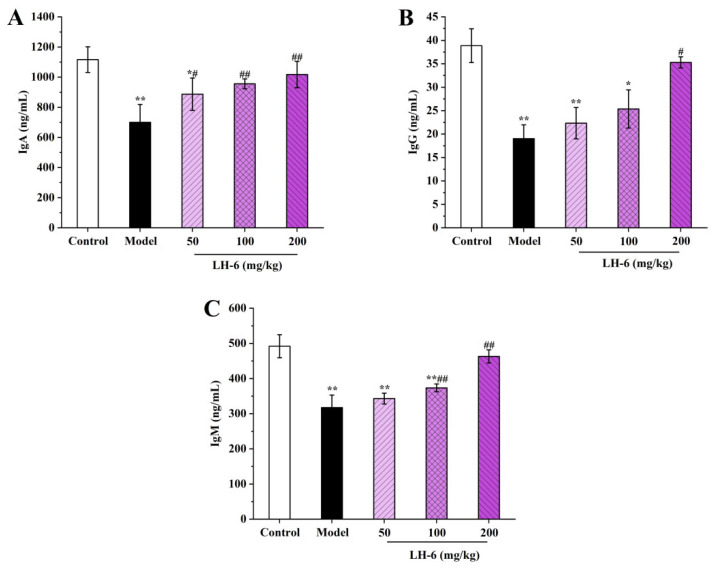
The effect of LH-6 on serum IgA (**A**), IgG (**B**), and IgM (**C**) levels in mice. 5 groups, *n* = 10. * *p* < 0.05 and ** *p* < 0.01 vs. control group; # *p* < 0.05 and ## *p* < 0.01 vs. model group.

Immunoglobulins secreted by B-lymphocytes are recognition units for specific antigens for mediating humoral immunity leading to protective functions [44]. As shown in Figure 8, immunoglobulin levels in the serum of the model group of mice were significantly declined compared to the control group (*p* < 0.01), indicating that CTX was able to inhibit immunoglobulin secretion. After treatment with LH-6, serum levels of IgA, IgG, and IgM were elevated to different degrees. Among them, the IgA, IgG, and IgM levels of 200 mg/kg LH-6 were 1017.51 ± 87.64, 35.29 ± 1.19, and 463.00 ± 18.71 ng/mL, respectively, which demonstrated no significant difference from those of the control group. This means LH-6 can improve the immunoglobulin level of mice induced by CTX and strengthen humoral immunity in mice.

### 3.9. Effects of LH-6 on Expression of NF-κB and MAPK Pathway-Related Proteins in Spleen

**Figure 9:**
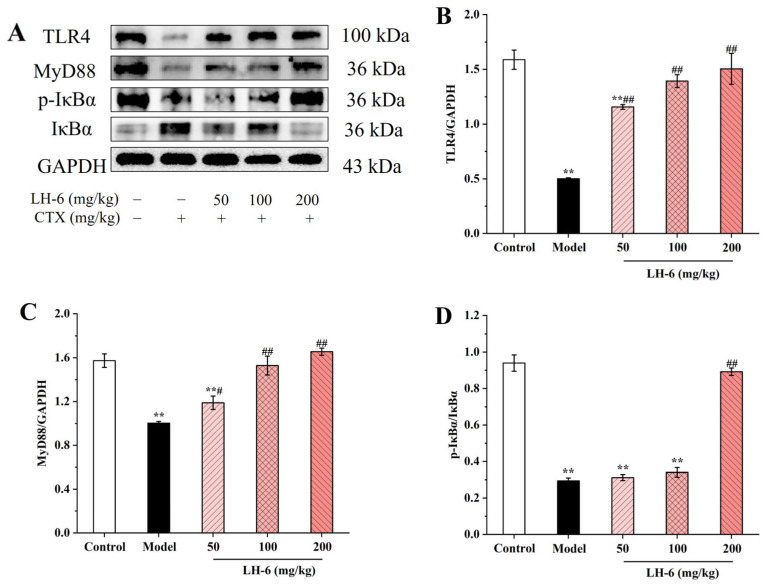
The effect of LH-6 on the expression of proteins related to the NF-κB signaling pathway in mouse spleen (**A**). TLR4 protein expression (**B**); MyD88 protein expression (**C**); p-IκBα protein expression (**D**); p-p65 protein expression (**E**). 5 groups, *n* = 10. ** *p* < 0.01 vs. control group; # *p* < 0.05 and ## *p* < 0.01 vs. model group.

The NF-κB signaling pathway induces the expression of pro-inflammatory genes and mediates the regulation of the inflammasome involved in multiple aspects of innate and adaptive immunity [45]. Figure 9 shows that compared with the control group, the expression of TLR4 and MyD88 proteins in the spleens of mice in the model group was significantly decreased (*p* < 0.01), and the phosphorylation of IκBα protein was also downregulated (*p* < 0.01). However, TLR4 and MyD88 protein expression as well as IκBα phosphorylation in the 200 mg/kg LH-6 group were notably above those of the model group, and were divided into 3.00-, 1.65-, and 3.03-fold those in the model group, suggesting that the LH-6-treated group suppressed the upregulation of the expression of these proteins. Our findings suggest that LH-6 activated the NF-κB pathway, thereby increasing the release of cytokines in the spleens of immunodeficient mice.

**Figure 10:**
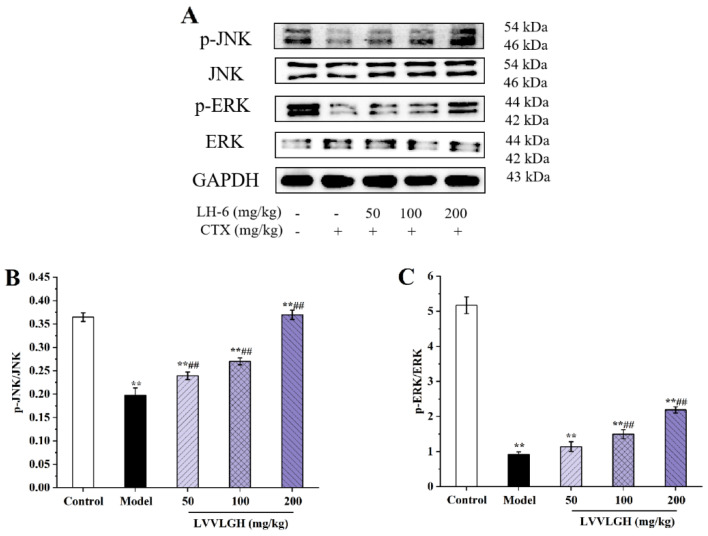
The effect of LH-6 on the expression of proteins related to the MAPK signaling pathway in mouse spleen (**A**). p-p38 protein expression (**B**); p-ERK protein expression (**C**). 5 groups, *n* = 10. ** *p* < 0.01 vs. control group; ## *p* < 0.01 vs. model group.

MAPKs, including JNKs, p38s, and ERKs, are mainly regulated by upstream MAPK kinases and MAPK phosphatases, and can trigger a series of cellular responses, such as proliferation, differentiation, and apoptosis [46]. Figure 10 illustrates the effect of LH-6 for MAPK pathway protein expression in the spleen of CTX-induced immunodeficient mice. After administration of LH-6, phosphorylated expression of JNK and ERK were prominently upregulated in a dose-dependent manner in the 200 mg/kg LH-6 group. Thus, LH-6 upregulates the phosphorylation level of spleen MAPK signaling pathway proteins in CTX-induced immunocompromised mice.

### 3.10. Molecular Docking Results of LH-6 with NF-κB and MAPK Pathways

**Figure 11:**
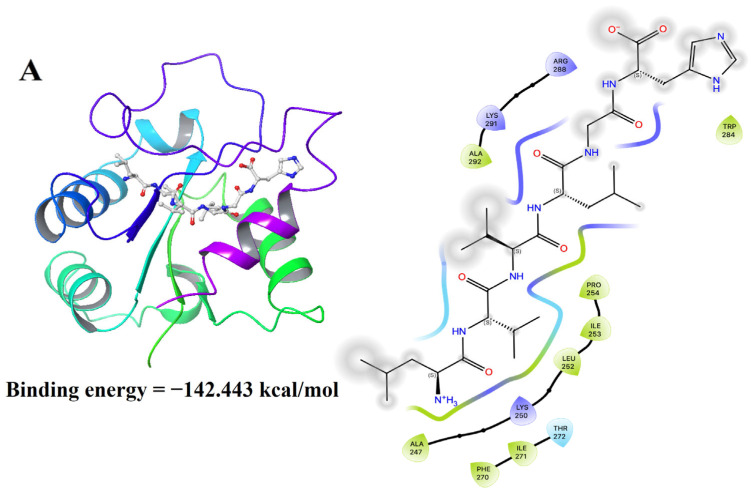

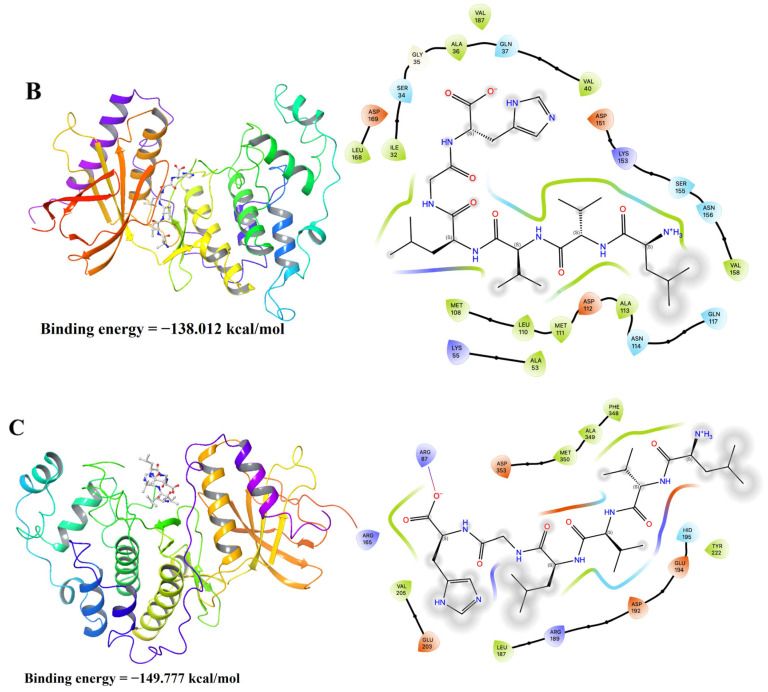
Molecular docking results of LH-6 with NF-κB and MAPK signaling pathways. MyD88 (**A**); JNK1 (**B**); ERK1 (**C**).

The docking energies of LH-6 binding to MyD88, JNK1, and ERK1 were −142.443, −138.012, and −149.777 kcal/mol, respectively, which indicates that the that the binding of LH-6 to NF-κB and MAPK was spontaneous [47]. Among them, LH-6 formed hydrophobic interactions with Ala247, Leu252, Ile253, and Pro254, and ionic bonds with amino acid residues Lys250 and Lys291 (Figure 11A). In addition, for the receptor protein JNK1, LH-6 formed hydrophobic interactions with Lle32, Met108, Met111, and Ala113 (Figure 11B). LH-6 formed a salt bridge with Arg87 of the receptor protein ERK1 and hydrophobic interactions with Ala349, Phe348, and Met350 (Figure 11C).

## 4. Discussion

Immunomodulation is a promising strategy that is a hot topic among researchers for the treatment of cancer, allergies, and autoimmune diseases [48]. CTX as an antitumor drug during chemotherapy can cause distress to the body, including weight loss, atrophy of immune organs, and immunodeficiency [49]. Therefore, amelioration of CTX-induced immunosuppression offers a reasonable rationale for the treatment of immunodeficiency diseases. In the current research, we explored the restorative effects of LH-6 on CTX-induced immunodeficiency in mice in terms of immunomodulation.

The thymus, which is composed of the cortex and the medulla, is critical for adaptive immunity, with a central role in the development and maturation of T-lymphocytes [50]. The spleen is the largest peripheral lymphoid organ involved in hematopoiesis, the removal of senescent erythrocytes, and the exertion of innate and acquired immune responses. Nevertheless, CTX causes disorder of the body’s immune system, which affects the responsiveness of primary and secondary lymphoid organs, including the thymus and spleen [51]. CTX not only inhibits lymphocyte proliferation but also atrophies immune organs, with a subsequent reduction in the spleen, the thymus, and body weight [52]. The results show that CTX disrupted the morphology of the thymus and spleen, blurred the boundary between red and white pulp in the spleen, intertwined the cortex and medulla in the thymus, and decreased the immune organ index. After administration, LH-6 amended the morphology of the thymus and spleen, with a significant increase in the organ index, suggesting that LH-6 could considerably modulate the CTX-induced immunocompromised state. Related reports, such as *Bacillus amyloliquefaciens*-derived nonapeptide (BAP), were similarly effective in mitigating the loss of immune organ indices and body weight in CTX treatment [32].

Macrophages, the first barrier to innate immunity, have the ability of phagocytosis, pathogen clearance, and antigen presentation [53]. When macrophages are in an activated state, they can synthesize NO for killing pathogens and enhancing phagocytic activity [54]. Therefore, phagocytosis and NO secretion are crucial characteristics of macrophages. NK cells, which are also part of the innate immune system, are capable of directly destroying target cells by releasing perforin and granzyme [55]. In this study, LH-6, to a certain extent, not only enhanced the phagocytosis and NO secretion of peritoneal macrophages but also enhanced the killing ability of NK cells on target cells YAC-1, indicating that LH-6 could improve the innate immunity of mice. Correspondingly, the fermentation product of *Pediococcus pentosaceus* has been reported to increase NO secretion in a manner that stimulates iNOS expression for the purpose of increasing macrophage immunity [56]. In addition, immune cells produce cytokines, including bioactive proteins of TNF-α, IL-1β, and IL-6, which modulate a variety of physiological responses, including inflammation and immune response [57]. In this study, LH-6 had the ability to upregulate the secretion of cytokines TNF-α, IL-1β, and IL-6 in CTX-treated mice to promote immunomodulation, but understanding of whether LH-6 modulates the associated DNA expression is still lacking and needs to be further demonstrated subsequently. In the future, we will use qPCR technology to determine gene expression regarding NF-κB or MAPK activation of transcription.

Acquired immunity mediated by T- and B-lymphocytes is usually divided into humoral and cellular immunity [58]. B-lymphocytes secrete specific immunoglobulins, IgG, IgM, and IgA, in response to pathogens, which are critical markers in the evaluation of humoral immunity [59]. Meanwhile, SRBCs as antigens also stimulate B-lymphocytes to generate corresponding antibodies [60]. Our study showed that LH-6 treatment notably increased serum IgG, IgM, and IgA, and serum hemolysin-specific antibody levels, illustrating that LH-6 restored CTX-induced immune dysfunction with enhanced humoral immunity in mice. Cell-mediated immunity is an immune response that involves the activation of T-lymphocytes and the killing of infected and tumor cells [61]. The DTH reaction is a typical manifestation of a T-lymphocyte-mediated type IV allergic reaction. The immunomodulatory effects of LH-6 on ConA-stimulated proliferation of splenic lymphocytes and delayed-type hypersensitivity reactions were investigated, which demonstrated that LH-6 had a proliferation-promoting effect on splenic lymphocytes and enhanced localized toe swelling, further elucidating that LH-6 enhances cellular immunity in mice. Above all, LH-6 synergistically promoted immunomodulatory activity in immunosuppressed mice by enhancing humoral and cellular immunity.

**Figure 12:**
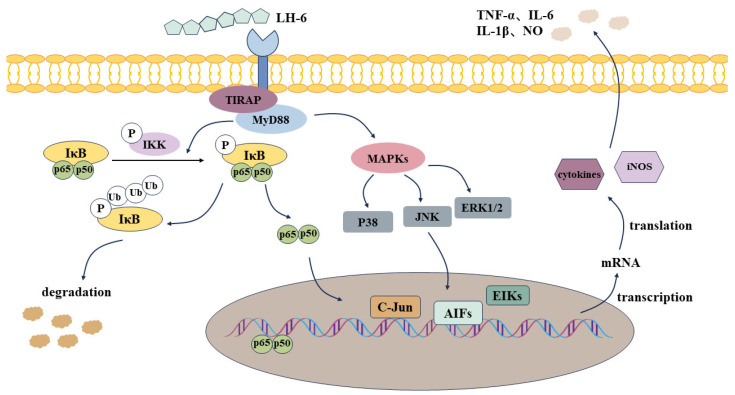
Potential immunomodulatory molecular mechanisms of LH-6 in immunosuppressed mice.

TLR4 is a transmembrane protein on the surface of various types of innate immune cells, such as macrophages, dendritic cells, and monocytes, and is extensively regarded as an essential receptor for the recognition of relevant stimulatory molecules [62]. Our study preliminarily showed that LH-6 may interact with the TLR4 receptor to activate the downstream NF-κB and MAPK signaling pathways. With the phosphorylation of IκBα, NF-κB protein dimers are released, and free NF-κB protein translocates into the nucleus and binds to DNA, regulating the transcriptional expression of pro-inflammatory genes that generate the release of cytokines [63,64]. MAPK, a mitogen-activated protein kinase, undergoes a tertiary kinase cascade of signaling upon stimulation by extracellular signals, initiating the degradation of NF-κB cascade proteins while transmitting signals to the nucleus, further promoting the secretion of pro-inflammatory factors (Figure 12). Meanwhile, the molecular docking results show that LH-6 could bind to the receptor proteins of NF-κB and MAPK with excellent binding energies, which further verified that LH-6 could spontaneously regulate and activate the signaling pathways of NF-κB and MAPK, and exerted immunomodulatory activities. However, limitations remain in this study. Although HPEPDOCK is applicable to peptides, docking alone does not confirm receptor involvement. Furthermore, direct receptor binding assays (e.g., SPR, MST, or receptor-blocking studies) were not performed in this study. In the future, we will perform receptor binding assays to improve the scientific rigor. Consequently, we draw the conclusion that LH-6 facilitated the activation of innate and acquired immunity by upregulating the expression of NF-κB and MAPK signaling pathways, ultimately achieving the goal of immunomodulation.

## 5. Conclusions

In summary, the present study investigated the in vivo immunomodulatory activity of LH-6 by establishing a mouse model of CTX-induced immunosuppression. The experimental results show that LH-6 ameliorated developmental disorders and the immune organ index as well as inhibited atrophy of the thymus and spleen in immunosuppressed mice. In particular, at a dose of 200 mg/kg, through activating the TLR4-dependent NF-κB/MAPK pathway, LH-6 contributed to the elevation in serum levels of inflammatory cytokines, which significantly promoted phagocytosis and NO secretion of abdominal macrophages, enhanced the killing activity of NK cells, and augmented the innate immunity of mice. Meanwhile, LH-6 promoted serum levels of IgG, IgM, and IgA as well as serum hemolysin; enhanced splenic lymphocyte proliferation and localized toe swelling; and synergistically enhanced humoral and cellular immune responses in mice. In addition, LH-6 can be employed as a promising functional food additive to enhance human immunity.

## Figures and Tables

**Table 1:** The effect of LH-6 on DTH induced by SRBC in mice (5 groups, *n* = 10).

Group	Initial/(mm)	Final/(mm)	Toe Swelling/(mm)
Control Model	2.45 ± 0.07 2.06 ± 0.08	2.96 ± 0.09 2.32 ± 0.06	0.51 ± 0.07 0.26 ± 0.03 **
Low-LH-6 Medium-LH-6 High-LH-6	2.29 ± 0.05 2.31 ± 0.09 2.27 ± 0.09	2.61 ± 0.11 2.68 ± 0.07 2.70 ± 0.17	0.32 ± 0.05 ** 0.37 ± 0.04 *# 0.43 ± 0.11 ##

* *p* < 0.05 and ** *p* < 0.01 vs. control group; # *p* < 0.05 and ## *p* < 0.01 vs. model group.

## Data Availability

The original contributions presented in this study are included in the article/Supplementary Materials. Further inquiries can be directed to the corresponding authors.

## Supplementary Materials

The following supporting information can be downloaded at: https://www.mdpi.com/article/10.3390/foods14111865/s1. File S1: The original data of H&E staining of mouse spleen and thymus and Western Blot.
