# Supplementary material for: Immunoenhancement Function of the Novel Hexapeptide (LVVLGH) from Thick-Shelled Mussel (Mytilus coruscus) on Immunodeficient Mice by Activating the NF-κB/MAPK Pathway
Source: Foods. 2025 May 24;14(11):1865. doi: 10.3390/foods14111865 (PMC12155323; doi:10.3390/foods14111865)

Figure 9A

TLR4

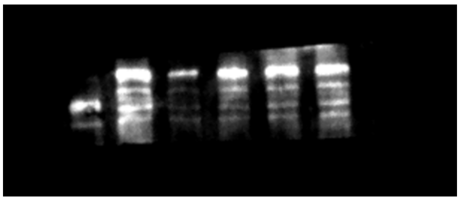

MyD88

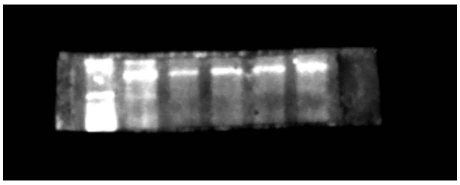

p-IκBα

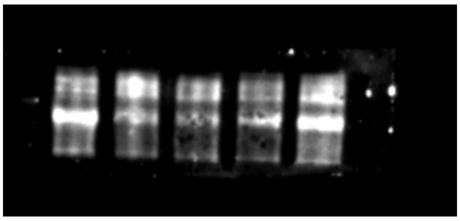

IκBα

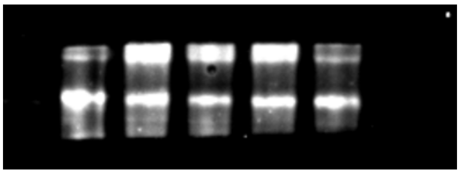

GAPDH

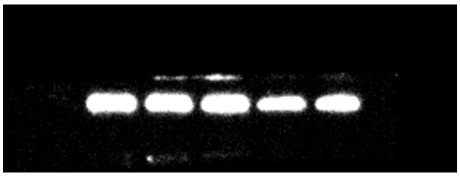

Figure 10A

p-JNK

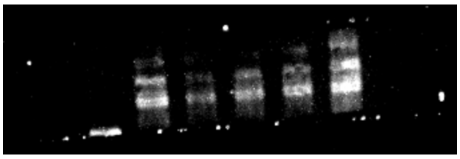

JNK

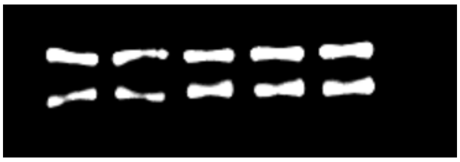

p-ERK

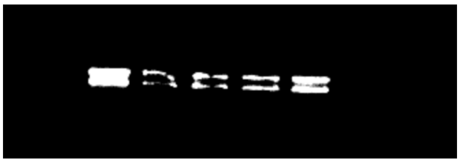

ERK

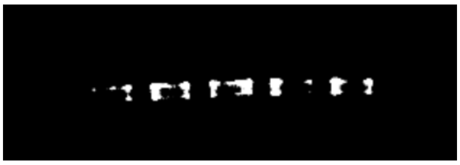

GAPDH

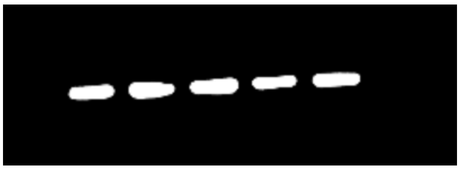

Supplement: Supplementary file 1 [file foods-14-01865-s001.zip › Western Blot/Figures 9A and 10A.zip!/Figures 9A and 10A.pdf]
